# Retraction: MicroRNA Let-7f-5p Promotes Bone Marrow Mesenchymal Stem Cells Survival by Targeting Caspase-3 in Alzheimer Disease Model

**DOI:** 10.3389/fnins.2026.2023227

**Published:** 2026-10-15

**Authors:** Frontiers Editorial Office

The journal retracts the 22 May 2018 article cited above.

Following publication, concerns were raised regarding the integrity of the images in the published figures. The authors failed to provide complete raw data or a satisfactory explanation during the investigation, which was conducted in accordance with Frontiers' policies. Given the concerns about the validity of the data, and the lack of complete raw data, this retraction was issued.

This retraction was approved by the Chief Executive Editor of Frontiers. The authors received a communication regarding the retraction and had a chance to respond. This communication has been recorded by the publisher.

Frontiers would like to thank Elisabeth Bik for contacting the journal regarding the published article.

